# Neuroprotection or Neurotoxicity of Illicit Drugs on Parkinson’s Disease

**DOI:** 10.3390/life10060086

**Published:** 2020-06-11

**Authors:** Carla Ferreira, Catarina Almeida, Sandra Tenreiro, Alexandre Quintas

**Affiliations:** 1 Molecular Pathology and Forensic Biochemistry Laboratory, Centro de Investigação Interdisciplinar Egas Moniz, P-2825-084 Caparica, Portugal; cferreira.lcfpem@egasmoniz.edu.pt (C.F.); catalmeida@gmail.com (C.A.); 2 Laboratório de Ciências Forenses e Psicológicas Egas Moniz, Campus Universitário–Quinta da Granja, Monte de Caparica, P-2825-084 Caparica, Portugal; 3 Faculty of Medicine of Porto University, Al. Prof. Hernâni Monteiro, P-4200–319 Porto, Portugal; 4 CEDOC–Chronic Diseases Research Center, Faculdade de Ciências Médicas, Universidade Nova de Lisboa, P-1150-082 Lisboa, Portugal; stenreiro@gmail.com

**Keywords:** Parkinson’s Disease, phytocannabinoids, amphetamine-type stimulants, novel psychoactive substances, cocaine, opioids

## Abstract

Parkinson’s Disease (PD) is currently the most rapid growing neurodegenerative disease and over the past generation, its global burden has more than doubled. The onset of PD can arise due to environmental, sporadic or genetic factors. Nevertheless, most PD cases have an unknown etiology. Chemicals, such as the anthropogenic pollutant 1-methyl-4-phenyl-1,2,3,6-tetrahydropyridine (MPTP) and amphetamine-type stimulants, have been associated with the onset of PD. Conversely, cannabinoids have been associated with the treatment of the symptoms’. PD and medical cannabis is currently under the spotlight, and research to find its benefits on PD is on-going worldwide. However, the described clinical applications and safety of pharmacotherapy with cannabis products are yet to be fully supported by scientific evidence. Furthermore, the novel psychoactive substances are currently a popular alternative to classical drugs of abuse, representing an unknown health hazard for young adults who may develop PD later in their lifetime. This review addresses the neurotoxic and neuroprotective impact of illicit substance consumption in PD, presenting clinical evidence and molecular and cellular mechanisms of this association. This research area is utterly important for contemporary society since illicit drugs’ legalization is under discussion which may have consequences both for the onset of PD and for the treatment of its symptoms.

## 1. Introduction

Neurodegenerative diseases are progressive incapacitating conditions involving the function loss of nerve cells in the brain or peripheral nervous system. These types of diseases affect millions of people worldwide, who may suffer an early death. The burden of neurodegenerative diseases has significantly increased worldwide over the past 25 years, mostly due to incremental increases in the population numbers, population ageing and to the environmental stress associated to contemporary societies [1,2,3]. These diseases have become the leading cause of disability and death in developed countries and, in spite of decades of research, there is no way to cure them or slow down their progression [4]. Neurodegenerative diseases include Alzheimer’s, amyotrophic lateral sclerosis, Huntington’s, Lewy body disease and Parkinson’s, among others. The two most prevalent neurodegenerative diseases are Alzheimer’s (AD) and Parkinson’s disease (PD). About 5% of people over the age of 85 suffer from PD, overwhelming health support systems and families [5]. The mechanisms underlying these particular conditions are related to the accumulation of misfolded and aggregated proteins, which may promote the disease either by a gain of the toxic activity or by loss of biological function [6,7,8,9]. The misfolded protein may aggregate into amyloid fibrils which deposit in the form of extracellular amyloid plaques, neuro-fibrillary tangles and other intracytoplasmic or intranuclear inclusions [8,9,10].

PD is currently the most rapid growing neurodegenerative disease [3,11] and, over the past generation, its global burden has more than doubled, as a result of human life span increment, better health conditions and environmental factors, such as exposition to the anthropogenic pollutant 1-methyl-4-phenyl-1,2,3,6-tetrahydropyridine (MPTP) and pesticides [11,12,13,14,15]. The onset of this disease usually occurs at near to average 60 years of age [16]. However, in some cases, PD takes place much earlier, between 21 and 50 years of age (early-onset PD (EOPD)) [17]. Symptomatically, initial phases of the disease are characterized by movement disorders, such as shaking, rigidity, slowness and aberrant gait [18]. Other symptoms, such as cognitive and behavioral problems, appear as dementia in the later phases of the disease [18]. The onset of PD and EOPD can arise as a consequence of genetic and/or environmental factors but most of the cases are sporadic [15,17,19,20,21,22,23]. It is generally accepted that known genetic causes may account for over 5% of the total PD population. A recent study was able to explain 16–36% of PD heritability, prevalence estimates (0.5–2.0%) [24]. The heritability in EOPD is higher than in later-onset PD [19]. These low values are in agreement with clinical reports describing the vast majority of PD cases as having an unknown etiology [3,11,17,25,26]. Several studies highlight that the combination of genetic and environmental factors, such as the consumption of substances and the variability of brain vulnerability, may also increase the risk to PD onset. These studies show that variability in genes associated with the cellular response and metabolism of xenobiotics or toxins increases the predisposition to develop PD [27]. Actually, the neurotoxic effect of MPTP changes the expression of genes associated with PD [28], which may alter the resilience of neurons to toxics [29,30]. Interestingly, scientific reports suggest that the incidence of PD and EOPD are increasing [11,17,31,32].

The onset of PD is tightly associated with the neuronal protein α-synuclein (α-syn). α-syn physiological function is to promote N-ethylmaleimide-sensitive factor (NSF) attachment to the soluble NSF attachment receptor (SNARE) protein complex assembly during synaptic exocytosis [33]. Thus, α-syn seems to regulate the synaptic vesicle release from presynaptic cells [33]. However, in pathological conditions, the overexpression and/or modification of α-syn forms neurotoxic aggregates which promote the selective loss of dopaminergic neurons in the *substantia nigra pars compacta* [34,35,36]. Most of the studies associate PD to several defected cellular and physiological mechanisms, such as neuroinflammation, excitotoxicity, mitochondrial dysfunction, reduced trophic support, abnormal kinase activity, disruption of calcium homeostasis and proteostasis dysfunction [37,38,39,40,41,42,43]. Nevertheless, there is still much to clarify, such as the precise molecular mechanism of amyloid fibrils formation, and its relationship with glial activation and peripheral immune cell infiltration in the inflammatory responses [7,44].

Recently, it has been hypothesized that the increase in the risk of PD might be associated with the consumption of drugs of abuse, such as stimulants [45]. The supporting pieces of evidence suggest that the neurotoxic effects of amphetamine-like stimulants on the nigrostriatal pathway are intriguingly similar to those in neurodegeneration observed in PD [46]. Furthermore, Parkinsonism was also described as a consequence of heroin consumption, which is a depressor of the central nervous system [47,48]. Conversely, scientific data supports that other substances of abuse, such as some phytocannabinoids, which interact with the endocannabinoid system, may play a neuroprotective role in PD [49,50,51,52,53,54,55]. Interestingly, both neurotoxic and neuroprotective effects are described as the outcomes from different drugs of abuse consumption. Finally, it is important to mention that during the last decade a new trend of synthetic molecules with similar effects to traditional illicit drugs, the novel psychoactive substances (NPS), has emerged and little is known about their toxicological impact. Consumption of NPS among young people may be a promotor of the initial stages of neurodegeneration and may well increase the future incidence of PD. Therefore, further studies with these substances are essential.

In this article, the current knowledge connecting illicit drugs of abuse and PD is reviewed and discussed.

## 2. Methodology

Searches were conducted in the online database *PubMed* and an advanced search were performed using the following boolean equations: (i) “misfolding diseases” AND “amphetamine”; (ii) “Parkinson” AND “amphetamine” with a cut-off filter to select just the papers from the last 10 years; (iii) “Parkinson” AND “phytocannabinoids” with a cut-off filter to select just the papers from the last 10 years; (iv) “Parkinson” AND “cathinone”; (v) “amyloid formation” AND “amphetamine” AND “Parkinson”; (vi) “heroin” AND “Parkinson”; (vii) “opioid” AND ”Parkinson”; (viii) “cocaine” AND “Parkinson”. The search was limited to English-language peer-reviewed journal publications. In the elimination process, papers that did not focus on illicit drugs of abuse or had no relationship between PD and illicit drugs of abuse were excluded. Further sources were identified by following up internal citations and references within the documents retrieved in the initial search.

## 3. Phytocannabinoids and Parkinson’s Disease

Phytocannabinoids are chemical substances present in *Cannabis sativa* and *indica*, commonly known as marijuana. Marijuana has been consumed for recreational, religious and medicinal purposes for at least five millennia [56]. Cannabis continues to be the most widely used drug worldwide [57]. United Nations Office on Drugs and Crime (UNODC) estimates that roughly 3.8% of the global population aged 15–64 years old used cannabis at least once during 2017 [57]. The cannabis is market as herb, resin and as hash oil [57]. The cannabis herb consists of the dried and crumbled leaves and flowering tops of the cannabis plant, which is generally smoked. In contrast, cannabis resin, the concentrated extract of cannabis flower and plant, is usually mixed with tobacco to be smoked [57]. Hash oil is a cannabis product which is extracted from any part of the plant using organic solvents [57]. Cannabis is controlled under the Single Convention on Narcotic Drugs of 1961 amended by the 1972 protocol [57]. Nowadays, the use of cannabis has been legalized in several countries. However, it remains illegal in the vast majority of the countries that have signed the UN convention on Narcotic drugs. Independently of its licit or illicit consumption, several studies point out medicinal benefits of products derived from marijuana for cases of glaucoma, neurodegeneration, multiple sclerosis, schizophrenia, cancer, epilepsy and eating disorders [58,59,60,61].

The two main cannabinoids present in marijuana, responsible for its psychoactive and medicinal effects, are ∆^9^-tetrahydrocannabinol (∆^9^-THC) and cannabidiol (CBD), respectively. The effects of ∆^9^-THC and CBD are mediated by receptors [62,63]. There are two known cannabinoid receptors subtypes, cannabinoid receptor 1 (CB1) and cannabinoid receptor 2 (CB2), which are also receptors for endogenous cannabinoids. The view of the endocannabinoid system as a therapeutic target has been bringing some researchers to explore potential defects in endocannabinoids metabolism, cannabinoids receptors and other components of the endocannabinoid system in PD onset [64,65,66,67].

### 3.1. Endocannabinoid System and Parkinson’s Disease

CB1 and CB2, encoded by cannabinoid receptor 1 and 2 genes, *CNR1* and *CNR2* genes, respectively. Both receptors exert biological effects by activating heterotrimeric Gi/o type G proteins, which lead to the inhibition of adenylyl cyclase and consequently to the reduction of cyclic AMP levels. These receptors also activate different members of the family of mitogen-activated protein kinases (MAPKs) [68]. The two receptors are differently distributed in the human body. Overall, CB1 distribution is more prominent in the central nervous system, whereas CB2 is mainly present in the immune system and, in a lesser extent, in the central nervous system [69,70]. CB1 has three isoforms from different expression CNR1 patterns. The full-length product of *CNR1* is mostly found in the brain and skeletal muscles, while the two isoforms are present in the liver and in pancreatic islet β-cells [69]. CB2 has also three isoforms, CB2A and CB2B resulting from alternative splicing of *CNR2*. The full-length CB2 is present in the central nervous system, mainly in glial cells, and in the immune system. The two isoforms CB2A and CB2B are predominantly expressed in testis and in the spleen [70].

Although several studies have shown the presence of CB2 in the brain, the role of CB2 in endocannabinoid-mediated synaptic transmission is still largely elusive [71,72]. However, it was reported that in medial prefrontal cortical pyramidal neurons, intracellular CB2 reduces neuronal firing through the opening of Ca^2+^-activated chloride channels, suggesting its involvement in the regulation of neuronal activity [73]. Moreover, CB2 receptors are involved in neuroinflammation by modulating microglia activation and migration [74] and are consequently associated with neurodegenerative disorders [75]. Accordingly, CB2 receptor is seen as a potential therapeutic target for PD since it modulates the inflammatory process and does not trigger undesirable psychoactive effects [76,77]. Furthermore, several shreds of evidence suggest that cannabinoid compounds may interact with other receptors, besides CB1 and CB2, namely the transient receptor potential cation channel subfamily V member 1 (TRPV1), the peroxisome proliferator-activated receptors (PPARs) and the G-protein-coupled receptor (GPCR) [78,79].

**Figure 1:**
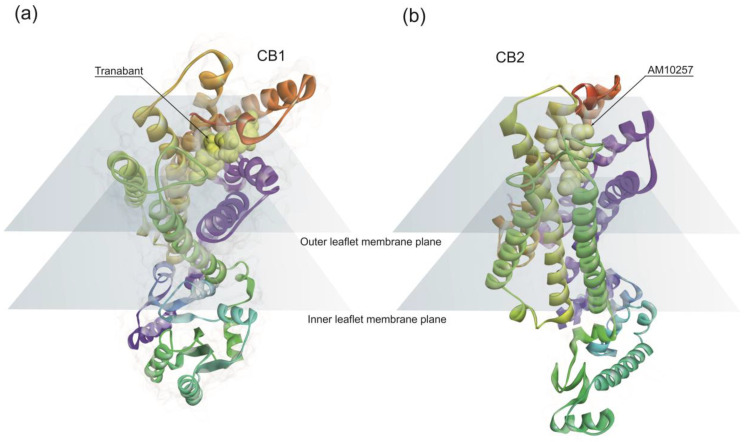
(**a**) Cannabinoids receptors 1 (PDB: 5u09) and (**b**) 2 (PDB: 6kpc). Helix 1 to 7 (red to purple) is mostly located inside the cell plasma membrane. (**a**) The cannabinoids receptor 1 (CB1) and (**b**) CB2 N-terminal loop (red) occupy the polar zone of the binding pockets. The CB1 receptor represented is bound to Tranabant, and CB2 receptor is bound to AM10257.

CB1 receptor structure has a classical 7TM fold (Figure 1a) similar to other rhodopsin family class A GPCRs [80]. Endocannabinoids bind to the CB1 hydrophobic binding pocket, activating the receptor. However, the position of the ligand-binding pocket of CB1 is different from the previously described binding sites of other class A GPCRs. Ligands lie low in the binding pocket of CB1, immediately above the conserved W356 [81]. Conformational changes in the surroundings of the residue W356 have been proposed as triggering CB1 activation [81].

CB2 has a high degree of homology with CB1, sharing 44% sequence identity [82]. The architecture of CB2 is also comprised of a 7TM fold (Figure 1b). The structural data indicates a critical role for the residue W258 as the toggle switch for CB2 activation [83]. Overall, structural data points out differences in the binding pockets between CB1 and CB2.

The endocannabinoid system plays an important role in central nervous system development and synaptic plasticity. This system is comprised of the endogenous cannabinoids, the cannabinoid receptors and the enzymes responsible for the synthesis and degradation of the endocannabinoids. N-arachidonoyl-ethanolamine or anandamide (AEA) was the first endocannabinoid found in the early 90s by Raphael Mechoulamt and its laboratory team [84]. Later, the same research group found 2-arachidonoylglycerol (2-AG), docosatetraenoyl ethanolamide (DEA) and noladin ether (2-AGE) [85,86]. Currently, about 15 endocannabinoids were found [87]. Endocannabinoids act on CB1, CB2, TRPV1, PPARS and several orphan receptors [87,88] and can be full agonists, partial agonists and/or antagonist, depending on the endocannabinoid and the receptor. For example, AEA is a high-affinity, partial agonist of CB1, and almost inactive at CB2 whereas 2-AG acts as a full agonist at both CBs with moderate-to-low affinity [89].

In the CNS, endocannabinoids are produced in post-synaptic neurons and act on presynaptic CB1, activating Ca^2+^ channels or decreasing neurotransmitter release by the vesicular release machinery [90,91]. 2-AG and anandamide are seen as ‘circuit breakers’ protecting glutamatergic neurons from excessive excitatory neurotransmission. Recently, it was suggested that the CB2 receptor mediates the reduction of excitability in the mouse ventral tegmental area [92]. In addition, several evidence are supporting endocannabinoid-mediated communication between neurons, astroglia and microglia [90,93,94]. In most cases, endocannabinoid-mediated retrograde signaling starts with the production of 2-AG, in response to increased intracellular Ca^2+^ concentration and/or activated Gq/11-coupled receptors [95,96]. 2-AG is then released into the extracellular space, via a mechanism not yet fully elucidated, and arrives at the presynaptic terminal where it binds to the CB1. Activated CB1 suppresses the release of neurotransmitter in two ways: first, by inhibiting voltage-gated Ca^2+^ channels, which reduces presynaptic Ca^2+^ influx; second, by inhibiting adenylyl cyclase (AC) and the subsequent cAMP/PKA pathway [95,96]. Signal termination requires the degradation of 2-AG by monoacylglycerol lipase (MAGL), which is expressed in selective synaptic terminals and glial cells [95,96]. The differential recruitment of 2-AG and AEA by several types of presynaptic activity has been described in the extended amygdala [97]. Moreover, AEA negatively regulates 2-AG metabolism in the striatum, the effect of which can be mimicked by the activation of TRPV1 [98].

Endocannabinoids play an important role in regulating basal ganglia physiology and motor function. Moreover, the modifications occurring in endocannabinoid signaling after dopamine depletion observed both in experimental models of PD and in patients with this condition, provide strong evidences for the involvement of the endocannabinoid system in PD. An abnormally high level of the AEA was found in 16 untreated patients who were diagnosed with PD [99]. It was suggested that the increase of AEA might be a result of a compensatory mechanism occurring in the striatum of PD patients, aimed at normalizing chronic dopamine depletion, thus extending for the first time to humans previous data on animal models of PD [99]. It can be explained since anandamide may inhibit the dopamine transporter function by a receptor-independent mechanism [100]. In untreated MPTP-lesioned primate, it was also found high levels of endocannabinoids, namely AEA and 2-AG in the striatum and 2-AG in *substantia nigra* [101]. Furthermore, anandamide can protect neurons from toxic insults such as glutamatergic excitotoxicity, nutrient deprivation, hypoxia, ischemia and apoptosis [102,103,104,105]. These protective effects of anandamide have been reported to be mediated by CB1 and CB2 cannabinoid receptors, whereas activation of TRPV1 has been suggested to mediate anandamide-induced apoptosis [106]. Several studies showed that treatment with anandamide lowered motor activity and produced hypothermia and analgesia in mice, increased inactivity time and markedly decreased ambulation and frequency of spontaneous non-ambulatory activities in rats [107,108]. The hypokinetic actions of AEA were boosted when co-administrated with a selective inhibitor of endocannabinoid uptake N-(3-furylmethyl) eicosa-5,8,11,14-tetraenamide, UCM707 [109]. A contradictory study showed that an intravenous administration of anandamide increased extracellular dopamine levels in the nucleus accumbens shell of awake, freely moving rats, a characteristic effect of most drugs of abuse in humans [110].

Levels and activities of AEA and 2-AG can be manipulated by inhibition of fatty acid amide hydrolase (FAAH) enzyme, the action of which is reduced in experimental models of PD [111,112]. However, it was evidenced that FAAH inhibition remarkably increases AEA tissue levels but reduces 2-AG levels [113]. The systemic administration of N-(4-hydroxyphenyl)-arachidonamide (AM404) enhances anandamide (AEA) availability in the biophase and exerts antiparkinsonian effects in 6-hydroxydopamine-lesioned rats. This is due to a reduction of D2 dopamine receptor function together with a positive modulation of 5-HT1B serotonin receptor function [114].

TRPV1 receptors also seem to play an important role in development and expression of dyskinesias in PD. The systemic administration of oleoylethanolamide, an agonist of PPARα and antagonist of TRPV1 receptors, reduces the development of dyskinesias dependent of a TRPV1-pathway in mouse model of PD, not involving PPARα receptors [115]. The intake of this compound induced the reduction of FosB striatal protein overexpression and the phosphoacetylation of histone 3, which are molecular markers of L-DOPA-induced dyskinesias. Actually, FOSB overexpression was previously associated with L-DOPA-induced dyskinesia in nitric oxide synthase-positive striatal interneurons in hemiparkinsonian mice [116]. This observation was correlated with the activation of ERK1/2 due to increased phosphorylation of its regulatory kinases [116]. It was suggested that GPR55 modulates anti-neuroinflammatory responses and movement control. These observations point to this receptor as a therapeutic target for the non-dopaminergic symptomatic treatment of PD [78,117].

The anti-inflammatory, antioxidant and proneurogenic properties of the endocannabinoid system make it a potential target to reduce the symptomatics of a number of neurodegenerative conditions [65,118,119,120]. Nonetheless, there are challenges in the development of drugs lacking psychoactive side effects and, to that end, targeting the anti-inflammatory non-psychotropic CB2 receptor is particularly promising [119,120,121].

There are shreds of evidence supporting the involvement of the endocannabinoid system in PD. Magnetic resonance imaging studies have shown regional differences in CB1 receptor availability in PD patients’ brains. According to one of these studies, CB1 availability was increased in mesolimbic and mesocortical regions of the brain, which are usually dopamine depleted in PD, and decreased in the *substantia nigra* [122]. Two other studies shown active involvement of CB1 in the regulation of L-DOPA action during PD therapy, preventing motor fluctuation through modulation of the striatonigral and striatopallidal pathway [123,124]. Regarding CB2, this receptor was found at significantly lower levels in tyrosine hydroxylase-containing neurons from *substantia nigra* of PD patients [125]. In contrast, studies in glial elements from post-mortem tissues of PD patients showed an increase in CB2 availability, either quantified by immunochemistry or by gene expression. These observations were then corroborated by studies in animal models [125,126,127,128]. The authors suggest that up-regulation of CB2 in glial cells is an indicator of the involvement of this receptor in neuroprotection.

### 3.2. Clinical Observations on Phytocannabinoids Use in Parkinson’s Disease

In countries where cannabis is legal, marijuana is used recreationally to self-medicate symptoms of disorders such as PD, multiple sclerosis, amyotrophic lateral sclerosis and schizophrenia [129]. About 44% of the population with PD is currently using marijuana [130]. In addition to cannabis, three cannabis derived products are also in use: dronabinol, nabiximols and nabilone [129]. Despite the lack of solid scientific evidence, PD patients using cannabis mention a positive impact on mood, memory, fatigue, obesity, sleep, pain, tremor, rigidity and bradykinesia after its consumption.

A study with 85 PD patients combined half a teaspoon of cannabis leaves, along with their prescribed pharmacotherapy for PD. About 46% of these individuals reported relief of PD symptoms on average 1.7 months after the first use of marijuana, suggesting chronic use of marijuana may be required for improvement in symptoms [131,132]. Overall, patients using cannabis have been reporting a lower level of disability after the intake of phytocannabinoids [130,133,134,135]. On the other hand, Carroll et al. have conducted a clinical trial with an orally administered cannabis extract which resulted in no objective or subjective improvement in dyskinesias or parkinsonism showing that results for clinical cannabis in PD still seem to be inconsistent [136]. Therefore, in determining if medical marijuana is beneficial as a PD therapeutic, some factors, such as chemical constituents, dose, delivery system and clinical outcomes, must be carefully controlled [137]. Cannabis is a complex plant with two main subspecies, namely *Cannabis sativa* and *Cannabis indica*, which can be differentiated by *C. indica* having higher cannabidiol content and *C. sativa* having a higher ∆9-THC content [138]. In addition, there are differences in ∆^9^-THC and CBD amount from strain to strain. Moreover, with the rising of the marijuana business, each sample might have different levels of ∆9-THC and CBD [139]. Furthermore, from the 538 natural compounds identified in *C. sativa*, more than 100 are phytocannabinoids. Therefore, the use of therapeutic cannabis is, surely a complex issue from the composition point of view [140,141].

Regarding the two most abundant phytocannabinoids found in *C. Sativa*, CBD showed to be the most promising, relieving some PD symptoms [142]. The first clinical studies with CBD pointed to a decrease in the psychotic symptoms [93] and significant improvements in measures of functioning and well-being of PD patients with no psychiatric comorbidities [94]. Overall, CBD shows significant therapeutic effects in reducing tremor, dyskinesia, rigidity and some non-motor symptoms, such as psychosis, rapid eye movement sleep behavior disorder, daily activities and stigma linked to relational and communication problems in PD [68,142,143,144]. However, larger-scale studies and randomized double-blind controlled studies are still needed to confirm the observations since several reports are mentioning negative effects [143].

A major concern with phytocannabinoids use is the inherent risk of PD patients to develop psychosis and cognitive impairment. This aspect makes them more susceptible to psychomimetic substances agonists of CB1, such as ∆^9^-THC or Nabilone [145,146]. In fact, it is well known that Nabilone may induce psychosis, even in patients without a psychiatric history [146]. Thus, patients with dementia should not be treated with agonists of CB1 to avoid further aggravation of neuropsychiatric symptoms [68]. Moreover, the PD patient’s personality must be taken into account to avoid the development of addictive behavior [147].

Overall, the evidence on the therapeutic use of medical marijuana and cannabinoid derivatives in patients with PD are heterogeneous and of poor quality [146]. Consequently, there is an urgent need for further scientific studies and to educate the caretakers on the pharmacology, known risks and known benefits of cannabis [148].

### 3.3. Studies on the Molecular and Cellular Mechanisms Underlying Clinical Observations

The positive clinical evidence observed in PD patients using medical marijuana and cannabinoid derivatives are leading researchers to address the cellular and molecular mechanisms underlying such outcomes. Therefore, a couple of studies suggest that molecules which bind to CB1 and/or CB2 receptors might be beneficial, since pharmacological modulation of the endocannabinoid system has been shown to reduce chronic activation of the neuroinflammatory response, reduce mitochondrial dysfunction and keep calcium homeostasis, resulting in the decrease of oxidative stress, which prevents the proapoptotic cascade, promoting neurotrophic support [59,122].

**Figure 2:**
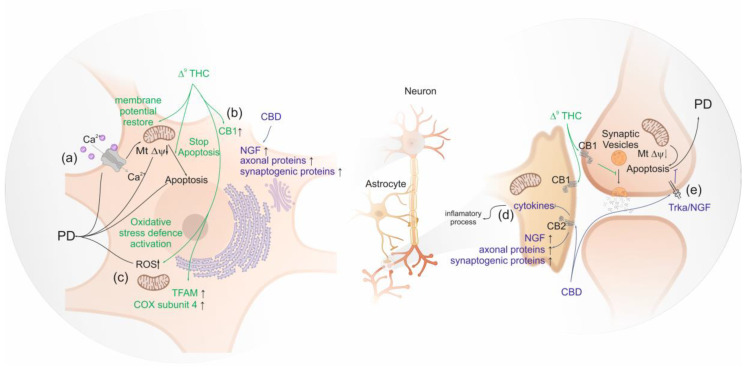
Neuroprotective mechanisms in Parkinson’s disease activated by phytocannabinoids. (**a**) The dysregulation of intracellular Ca^2+^ levels result in excitotoxicity which results in mitochondrial depolarization. CB1 activation restores membrane potential; (**b**) Activation CB1 promotes gene expression of cannabinoid receptor 1 (*CNR1*) reducing neurotransmitters release; (**c**) Increasing levels of reactive oxygen species (ROS) promote the formation of protein toxic oligomers. CB1 activation decrease ROS levels by expressing mitochondrial transcription factors (TFAM) and restoring mitochondrial DNA levels; (**d**) CB2 activation decrease pro-inflammatory cytokines release; and (**e**) CB2 activation inhibit apoptosis by nerve growth factor receptor (NGF) also known as Tropomyosin receptor kinase A (TRKA).

Studies in human differentiated neuroblastomas, which have dopamine beta-hydroxylase activity, show that Δ^9^-THC seems to be neuroprotective by up-regulating the expression of gene encoding CB1, suggesting a direct neuronal protective effect of Δ^9^-THC mediated via PPARγ not involving CB2 [55]. The activity of dopamine beta-hydroxylase modulates the levels of dopamine [149]. Busquets-Garcia et al. (2016) observed that normal circulating adrenaline and noradrenaline levels are sustained after stress by AM6545 pre-treatment, a full agonist of CB1 [150]. Thus, the clinical observation that the increment of CB1 availability in mesolimbic and mesocortical regions of brain seems to be neuroprotective [122]. Moreover, the involvement of PPARγ activation in the neuroprotective effect of Δ^9^-THC is also suggested, as it induces the transcription of proteins involved in oxidative stress defense and mitochondrial biogenesis, promoting mitochondrial normal function in PD [151]. In addition, the reduction of oxidative stress was linked to the restored the Peroxisome proliferator-activated receptor-gamma coactivator (PGC-1α) levels which regulate energetic metabolism [151]. In fact, low basal levels of PGC-1α are expected to be associated with enhanced glycolytic metabolism, low oxygen consumption and elevated reactive oxygen species (ROS) levels [152]. In addition, the observed ∆^9^-THC mitochondrial biogenesis may be linked to its ability to induce the mitochondria transcription factors (TFAM) expression and to restore mitochondrial DNA levels leading to increased cytochrome c oxidase subunit 4 (COX4) [151], the terminal enzyme complex of the respiratory chain which is linked to PD [153] (Figure 2).

A high concentration of glutamate induces deregulation of intracellular Ca^2+^ levels which results in mitochondrial Ca^2+^ overload and membrane depolarization, triggering the mechanism of cell death [94]. Δ^9^-THC also seems to play a neuroprotective effect against glutamate-induced neurotoxicity, in neural primary cells, by restoring mitochondrial membrane potential which produces an anti-apoptotic effect. In the same study, a decrease in the levels of glutamate was observed, which in turn decreases capase-3 levels, one of the critical enzymes of apoptosis. Overall, CB1 activation by Δ^9^-THC seems to slow down the degenerative processes in PD associated with the overflow of glutamate [154].

Cannabidiol also presents a neuroprotective activity against MPP^+^, a neurotoxin which triggers PD, by the activation of nerve growth factor receptor (NGF) also known as Tropomyosin receptor kinase A (TRKA), and the increment in the expression of axonal and synaptogenic proteins [155]. Other compounds found in *Cannabis sativa*, such as β-caryophyllene and Δ^9^-tetrahydrocannabivarin (Δ^9^-THCV), showed the potential to prevent the onset of PD. β-caryophyllene activates CB2, leading to a decrease of oxidative/nitrosative stress, to a decrease of pro-inflammatory cytokines release and to an inhibition of gliosis, which reduces neuroinflammation and nigrostriatal degeneration [156,157]. Δ^9^-THCV is a potent CB2 receptor partial agonist in vitro and it antagonizes cannabinoid receptor agonists in CB1-expressing tissues. However, in vivo Δ^9^-THCV behaves both as an antagonist or, at higher doses, an agonist of CB1 [58]. It has been shown that acute administration of this phytocannabinoid attenuated the motor inhibition caused by changes in glutamatergic transmission, and the chronic administration of Δ^9^-THCV has reduced the loss of tyrosine hydroxylase–positive neurons caused by 6-hydroxydopamine in the *substantia nigra* [158] (Figure 2).

In general, the effects of some phytocannabinoids on PD appear to be protective either by binding to the CB1 receptor, inhibiting dopamine beta hydroxylase activity and decreasing glutamate levels or by binding to CB2, reducing neuroinflammation.

### 3.4. Is There Enough Data Supporting Protective or Therapeutic Role of Cannabinoids on PD?

Overall, clinical observations and research outcomes support the endocannabinoid system as a target to alleviate the symptoms of PD. Actually, patients using cannabis have been reporting a lower level of disability after the intake of phytocannabinoids [130,133,134,135]. However, the evidence on the therapeutic use of cannabinoids in patients with PD are heterogeneous and of poor quality [146]. At molecular and cellular levels, the evidence is promising for the use of phytocannabinoids in PD. Phytocannabinoids reduce neuroinflammatory response, mitochondrial dysfunction and oxidative stress [59,122]. Additionally, Δ^9^-THC plays a neuroprotective effect against glutamate-induced neurotoxicity, in neural primary cells slowing down neuron degeneration due to overflow of glutamate [154]. Epidemiological studies are also encouraging. A retrospective survey found an improvement of PD symptoms with medical cannabis in the initial stages of treatment, with no evidence of major adverse effects [49]. Another epidemiological study pointed to the possible effect of cannabidiol in improving the quality of life of PD patients without psychiatric comorbidities. However, the authors found no statistically significant differences concerning the motor symptoms of PD [159].

Despite the shreds of evidence suggesting that the consumption of cannabinoids can reduce PD symptoms, some authors argue that there are not enough studies for such a conclusion [50,51,52,53,54]. Stampanoni Bassi et al. (2017) concluded that results from available clinical studies are controversial and inconclusive due to several limitations, including small sample size, lack of standardized outcome measures and expectancy bias [54,160]. They propose studies involving a larger sample of patients, appropriate molecular targets, objective biological measures (i.e., cannabinoids blood level) and specific clinical outcome measures to clarify the effectiveness of cannabinoids-based therapies [54]. Moreover, most of the studies investigating the therapeutic potential of cannabinoids in PD have been conducted in animal models, and an insufficient number of clinical trials have been carried out. Furthermore, the therapeutic benefits demonstrated in animal models will require further study in humans avoiding extrapolation between them, since animal models may not properly induce or recapitulate PD pathology [51,52,119,148,161,162,163,164]. Thereby, in the present, the studies investigating the role of phytocannabinoids are few and limited to understand its beneficial effects. The improvements needed for further successful research in this area are (i) larger sample size; (ii) well-designed studies testing cannabis in PD patients population to establish evidence-based data on the scope of pharmacological benefits and adverse effects; (iii) long term evaluation of disease progression; (iv) identification of the precise formulation for each type of pathology and each subset of patients for achieving a neuroprotective effect [51,52,119,148,162,163,164]. Overall, there is a clear need for further studies in humans.

## 4. Amphetamine-Type Stimulants and Parkinson’s Disease

According to the World Health Organization, amphetamine-type stimulants is a group of drugs of abuse whose principal members include amphetamine and methamphetamine [165]. Amphetamine was firstly synthesized in 1887 in Germany as phenylisopropylamine by Romanian chemist Lazăr Edeleanu [166]. Methamphetamine was synthetized in 1893 by Nagayoshi from ephedrine [167], an alkaloid present in the plant Ephedra, isolated for the first time in 1885 by G. Yamanashi and named by Nagai in 1887 [168]. Amphetamine-type stimulants have been used for recreational purposes to improve physical and mental performance in fatigued subjects. During World War II, amphetamine and methamphetamine were used extensively by Allied and Axis forces for their stimulant and performance-enhancing effects [169].

As the addictive properties of the drugs became known, governments began to place strict controls on the sale of the drugs. As a result of the United Nations 1971 Convention on Psychotropic Substances, amphetamine became a schedule II-controlled substance, as defined in the treaty, ratified by all 183 state members at the time. Despite strict government controls, amphetamine and methamphetamine are used for recreation purposes, and according to the European Monitoring Centre for Drugs and Drug Addiction (EMCDDA), amphetamines are associated with a large number of health emergencies in the north and east of Europe [170]. The monitoring center estimates that 1.2 million of European young people between 15–34 age have consumed amphetamine-type stimulants in the last year, and 12.4 million of European people have consumed them somewhere during their lifetime [170].

**Figure 3:**
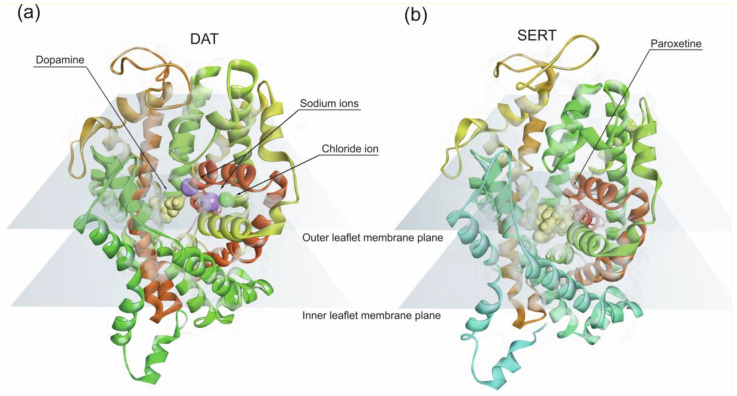
(**a**) Dopamine transporter (PDB: 4xp1) and (**b**) serotonin transporter (PDB: 6vrh). Both dopamine transporter (DAT) and 5-HT transporter (SERT) have twelve transmembrane (TM) helices arranged in a barrel-like bundle, connected by 5 intra- and 6 extracellular loops. The substrate binding site is located in the core of the protein structure. Dopamine receptor represented is bound to dopamine and the serotonin receptor to paroxetine.

Amphetamine-type stimulants share structural features with the catecholamine neurotransmitters, such as noradrenaline and dopamine with twelve transmembrane (TM) helices arranged in a barrel-like bundle [171]. Amphetamine-type stimulants have an aromatic ring and a nitrogen on the aryl side-chain which is a prerequisite for competitive binding to the monoamine reuptake transporters, noradrenaline transporter (NET), dopamine transporter (DAT) and 5-HT transporter (SERT) [171]. All three transporters are membrane-embedded proteins (Figure 3) expressed in the presynaptic neuronal terminals. Monoamine reuptake transporters mediate the uptake of neurotransmitters from the synaptic cleft, into the pre-synaptic neuronal terminals using the energy gradient produced by Na^+^/K^+^ ATPase. DAT and NET translocation of dopamine and norepinephrine involve co-transport of two Na^+^ and one Cl^−^ ion along with one molecule of substrate. SERT co-transports one 5-HT molecule with one Na^+^ and one Cl^−^ along with one K^+^ ion in the opposite direction.

Since amphetamine competes with endogenous monoamines for transport into the nerve terminals via these transporters, the higher the concentration of amphetamine present in the synapse, the less molecules of endogenous catecholamines are uptake due to competitive inhibition of DAT by amphetamine. Consequently, there is a greater stimulation effect on postsynaptic receptors by dopamine [171]. Amphetamine also has an affinity for vesicular monoamine transporter 2, preventing the translocation of monoamines into the intraneuronal storage vesicles and reversing the direction of the reuptake transporter. Therefore, it pumps neurotransmitters out of neurons into the synapse [94,172]. In addition, amphetamine also increases synaptic monoamine concentrations inhibiting monoamine oxidase, which catalyzes the breakdown of monoamine neurotransmitters in the CNS.

The abuse of amphetamines-type stimulants has been largely described as affecting dopaminergic transmission and function, inducing dopamine depletion, rising extracellular dopamine levels and prolonging dopamine receptor signaling in the striatum. The consequences of amphetamines-type stimulants intake have been suggesting a relationship between its consumption and the onset of PD [173,174]. The studies performed with amphetamine and methamphetamine show that these two substances have similar pharmacokinetic profiles and their dopamine responses in the striatum are equivalent [175]. Some studies tried to verify the amphetamine-like stimulants effects in PD symptoms treatment, however no significant improvement has been found [176].

### 4.1. Clinical Observations of Amphetamine-Type Stimulants Use in Parkinson’s Disease

Amphetamine-type stimulants effects are euphoria, mood elevation, sense of wellbeing, energy, wakefulness, fatigue decrease, focus and alertness increase [177,178,179]. Repeated administration of amphetamine-type stimulants leads to neuroadaptation and impaired basal functioning, which can result in a depressed mood, cognitive impairment, leakage of the blood-brain barrier by hypoperfusion in the striatum, causing hypoxia and dopamine reduction [180,181,182]. Chronic methamphetamine use causes neurotoxicity, damaging the dopamine neurons in the nigrostriatal pathway, due to a rise in α-syn levels in *substantia nigra*, which may increase the risk of developing PD in later life [180,183,184,185]. In addition, it has been observed that the intake of these substances during mice adolescence may later increase their vulnerability for neuroinflammation and cell death by toxins, such as 1-methyl-4-phenyl-1,2,3,6-tetrahydropyridine (MPTP) [186]. Over the years, these damaged cells may die precociously, depleting the reserve of neural cells necessary for normal neurological function and, when a critical number of cells are lost, parkinsonism starts developing [183]. In addition, neurotoxic doses of methamphetamine cause depletion in the dopamine content of striated tissue. This depletion should also be considered as a clinical consequence to the brain, regardless of the absence of neuronal loss or physiological nerve changes [187]. Thereby, amphetamine-type stimulants make dopamine pathways, involved in motor function and limbic-motor integration, vulnerable to progressive degeneration increasing the predisposition to PD [188].

### 4.2. Studies on the Molecular and Cellular Mechanisms Underlying Clinical Observations

Protein misfolding and aggregation processes are involved in several neurodegenerative diseases and are a consequence of conformational changes in the amyloid protein precursors. In PD, α-syn aggregates form amyloid deposits in the brain called Lewy bodies, which are associated with the loss of dopaminergic neurons in the *substantia nigra*. Thus, some researchers are studying the relationship between α-syn and amphetamine-type stimulants, such as conformational changes, post-translational modification and increased protein expression [189,190,191]. Amphetamine and methamphetamine bind tightly to N-terminus of intrinsically unstructured α-syn inducing a folded conformation. A putative fold conformation increases the likelihood of misfolding and aggregation. Consequently, the authors suggest that this mechanism may increase the incidence of PD amongst amphetamine and methamphetamine users [189,190]. Additionally, Wang et al. (2014) suggested an increment of α-syn levels due to methamphetamine-induced excessive heat. Actually, the temperature in the mid-brain region can exceed 41 °C upon ingestion of this stimulant [191]. Repeated bouts of excessive heat increase α-syn expression to prevent cells from heat damage by inhibition of stress signaling. Consequently, this causes an accumulation of α-syn promoting its aggregation, which in turn damages neurons [191]. Moreover, post-translational modifications of α-syn, such as phosphorylation, nitration, acetylation and ubiquitination, have also been pointed as a risk or beneficial factor for PD [192,193,194,195]. However, only protein nitration has been linked to the use of methamphetamine, which is pointed out as a risk due to the increased post-translational modifications of α-syn which seems to mediate neurotoxicity, as judged by studies in human neuronal lines and mice brain cells [192].

Methamphetamine influences gene expression of normal dopaminergic innervation in striatum via stimulation of dopamine and glutamate receptors [196,197,198]. Low doses of methamphetamine were also found to induce the expression of a different set of genes in lesioned denervated striatum, completely lacking dopamine. These observations implicate an alternative gene expression activation independent from dopamine in the presence of methamphetamine [196]. In addition, the authors suggest that the absence of dopamine might cause plastic changes that render the striatum differentially responsive to the effects of methamphetamine [196]. Another study observed the neurotoxic effects of methamphetamine in rodent models using epigenetics assays, showing that the consumption of this substance decreased cytosine methylation in SNCA promoter region, and consequently upregulates α-syn in *substantia nigra,* contributing to the Parkinson’s-like behavior [199].

**Figure 4:**
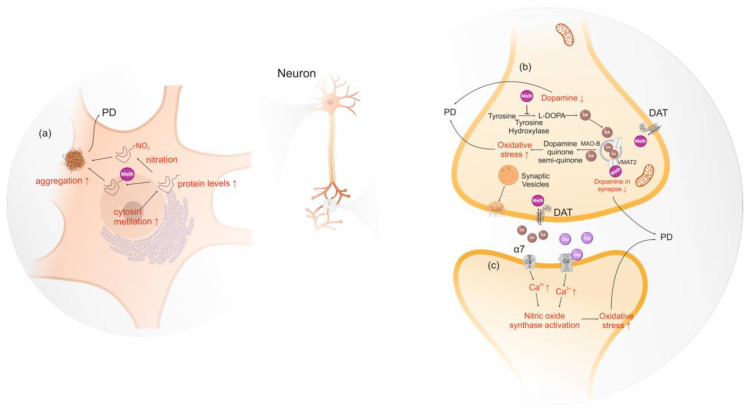
Parkinson’s disease neurotoxic pathways triggered by amphetamine-type stimulants. (**a**) Amphetamine-like stimulants can promote the formation of protein aggregates by (i) increasing the α-syn level; (ii) bind tightly to N-terminus of intrinsically unstructured α-syn adopting a folded conformation; (iii) post-translational modification of α-syn by nitration; (**b**) decrease the dopamine levels; (**c**) generation of ROS by (i) dysregulated cellular Ca^2+^ which activate nitric oxide synthetase or (ii) dopamine oxidation.

Regarding the cellular mechanisms underlying the neurotoxic effects of amphetamine-type stimulants, these substances activate nicotinic alpha-7 receptors, which increase intra-synaptosomal calcium, nitric oxide synthase and protein kinase C, leading to the production of high levels of nitric oxide and to dopamine oxidation, which promotes neurodegeneration [200]. The increase of nitric oxide synthase may modulate fundamental functions since nitric oxide is involved in almost all vital functions, from platelet aggregation to neurotransmission [201]. Cells treated for 24 h with methamphetamines significantly increased its nitric oxide synthase, causing a rise in nitric oxide and α-syn levels, that consequently promoted the aggregation of α-syn [202]. Another study has also suggested that tyrosine hydroxylase, dopamine transporter, vesicular monoamine transporter 2, nitric oxide synthase and reactive oxygen species may be involved in α-syn mediated methamphetamine-induced neuronal toxicity [203] (Figure 4).

Oxidative stress-induced by amphetamine-type stimulants is also linked to PD since it increases dopamine neurons vulnerability. A study in pregnant primates exposed to methamphetamine showed that high levels of oxidative stress in pregnancy can compromise the population of nigrostriatal dopamine neurons and potentially elevate the risk of PD in the born child’s later life [204]. It was proposed that the higher levels of oxidative stress, induced by amphetamine-like stimulants, are a consequence of dopamine autoxidation which increases excitotoxicity [205]. However, there are also evidences that the exposure to low levels of methamphetamine induces a certain degree of cellular stress that can reduce the vulnerability of dopamine neurons to insults. The activation of a small stress response can be used to protect neuron against neurodegeneration and might be used pharmacologically [196]. The cellular mechanisms underlying stress-induced protection are associated to (i) decrease of basal ERK 1/2 and kinase b levels, involved in multiple cellular processes such as apoptosis; (ii) reduced activity of protein phosphatase 2, a protein phosphatase implicated in ERK1/2 dephosphorylation, inhibiting it; and (iii) upregulation of the pro-survival protein BCL-2, which plays an anti-apoptotic role [196].

### 4.3. Is There Enough Data Supporting a Neurotoxic Role of Amphetamine-Type Stimulants on PD?

Overall, clinical observations point out amphetamine-type stimulants as neurotoxic. These substances damage dopaminergic neurons, involved in motor function and limbic-motor integration, increasing the predisposition to PD [188]. The molecular studies show that amphetamine upregulates α-syn in *substantia nigra* which accumulates leading to aggregation, which in turn damages neurons [191] contributing to the Parkinson’s-like behavior [199]. Conversely, there is evidence that exposure to low levels of methamphetamine may reduce dopamine neurons vulnerability to insults. Epidemiological studies suggest an increased risk of PD for amphetamine-type stimulants users independently of the lifestyle [45,206,207,208]. In fact, a nearly 3-fold increased risk of PD in amphetamine-type stimulants users vs. non-consumers was described [209]. Moreover, a retrospective case-control study revealed that prolonged use of amphetamines is associated with 8-fold increased risk of PD, with an average of 27 years between amphetamine exposure and the onset of disease signs [183].

Despite this epidemiological evidence, some studies suggest that there is not enough data to indicate that amphetamine-type stimulants exposure causes loss of dopamine neurons in humans, and consequently the appearance of PD [187,210]. In some consumers, the exposure to methamphetamine resulted in dopamine loss, more marked in caudate than in putamen, whereas in PD the putamen is distinctly more affected [187,210]. However, striatal dopamine deficiency is evident in methamphetamine consumers which are explained by a loss of dopamine in intact neurons and/or loss of dopaminergic neurons. According to the authors, this can be partially resolved by dopamine substitution medication in some individuals [210].

Other studies agree that these drugs may not directly evoke PD, but might predispose the central nervous system for Parkinson-like syndromes in long-term exposure [174,211]. Perfeito et al. (2013) showed the evidence of neurotoxic events linked to dopamine-induced oxidative stress and decreased protein quality control and Volkow et al. (2015) showed an acceleration of the age-related loss of dopamine neuronal function [174,211]. Therefore, the use of amphetamine-type stimulants may be an initiating event in the development of PD and parkinsonism, in conjugation to other risk factors that a given individual may hold [212]. Corroborating that the interplay of genetic and environmental risk factors increases the susceptibility to sporadic PD, a recent study found a significantly higher allele and genotype frequency of the CYP2D6*4 variant in 174 sporadic PD patients when compared to 200 controls [27] providing evidence on the hypothesis that a poor metabolizer status may increase the risk to develop PD especially in populations that are exposed to environmental toxins [27].

## 5. Cocaine

Cocaine is extracted from leaves of two distinct species of the genus *Erythroxylum* (family Erythroxylaceae): *Erythroxylum coca* Lam. and *Erythroxylum novogranatense* (Morris) Hieron [213]. Coca leaves chewing is part of the Andean lifestyle for thousands of years. At the end of the 19th Century, pharmaceutical and food products with coca leave extracts were introduced in the market achieving high popularity. Later, the active principle present in coca leaves was purified and used in medicine both as a stimulant for psychanalysis and as an anesthetic. Simultaneously, the use of pure cocaine for recreative purposes also started. Nowadays, the cocaine market is the second-largest illicit drug market in the EU, after cannabis [214]. According to the EMCDDA 2019 drug report, about 4 million people in the EU have used cocaine in 2018 [214].

Cocaine acts on presynaptic monoamine reuptake transporters inhibiting monoamine neurotransmitters reuptake which increases its levels in the synaptic cleft [215,216]. The atomic structure of dopamine transporter of *Drosophila melanogaster* bound to cocaine was obtained in 2015 [217]. This structure was used as a template in combination with computational tools to study the binding and modulation of human dopamine transporter function by dopamine and cocaine. This study showed that cocaine competitively binds dopamine transporter. However, the binding affinity is dependent on the conformational state of dopamine transporter [216]. Notwithstanding, cocaine binds competitively to dopamine transporter inhibiting dopamine reuptake [218].

### 5.1. Clinical Observations of Cocaine Use in Parkinson’s Disease

Cocaine exposure may have neurotoxic effects on dopaminergic neurons since the total number of melanized dopamine cells in the anterior midbrain is reduced in cocaine users [219]. In addition, chronic cocaine use leads to down-regulation of post-synaptic dopamine receptors which results in putamen hypertrophy as a compensatory process to produce more dopamine to maintain dopaminergic transmission [220,221]. However, in PD brains, both caudate and putamen volumes were smaller when compared to controls [222,223]. On the other hand, there is a published case report of a young adult that developed early parkinsonism after chronic cocaine use [224]. To date, the effect of cocaine use in PD is still controversial.

### 5.2. Studies on the Molecular and Cellular Mechanisms

In a similar way to amphetamine, cocaine binds tightly to N-terminus of intrinsically unstructured α-syn, inducing a folded conformation, which increases the likelihood of misfolding, and possibly leading to an increased incidence of PD amongst drug users [189]. Moreover, cocaine has been shown to increase the levels of α-synuclein [225,226,227]. A recent genetic study links the cocaine abuse to secondary Parkinsonism as a consequence of a potential gene-environmental interaction, namely a detected leucine-rich repeat kinase 2 (*LRRK2*) risk variant [224].

### 5.3. Is There Enough Data Supporting a Neurotoxic role of Cocaine on PD?

Despite the scarcity of the clinical and bench research data on cocaine and PD, several studies have shown that cocaine is not a risk factor for PD onset [45,228,229,230]. There is a consensus that high levels of cytosol dopamine are neurotoxic. It was observed that after cocaine administration the cytosol levels of dopamine remained unchanged suggesting that cocaine administration may not be considered a risk factor in terms of dopamine-induced neurodegeneration [228]. This is reinforced by the observations that cocaine enhances dopamine levels in the dorsal, but not in ventral, striatum [229]. Finally, a three-day administration via implanted minipumps of cocaine hydrochloride did not produce axonal degeneration in the frontal agranular cortex or neostriatum [230]. Interestingly, cocaine has been shown to alleviate the symptoms of PD in monkeys [231].

## 6. Opiates and Parkinson’s Disease

Opiates comprise the naturally occurring alkaloids found in the opium poppy from the plant *Papaver somniferum*, such as morphine, codeine and also their semi-synthetic derivatives, heroin, hydrocodone, oxycodone and buprenorphine among others [232]. Most pharmaceutical opioids are controlled under the Single Convention on Narcotic Drugs of 1961 with some exceptions, such as buprenorphine, which are controlled under the Convention on Psychotropic Substances of 1971. The prevalence of opiates consumption in Europe in 2017 was estimated at 0.7% of the adult population, representing nearly 3.8 million opioid users. In Western and Central Europe, where there are an estimated 2 million opioid users (0.6% of the adult population), the use of opioids is dominated by heroin [233]. In addition to heroin, the most common opioids are opium, morphine, methadone, buprenorphine, tramadol and various fentanyl analogues.

**Figure 5:**
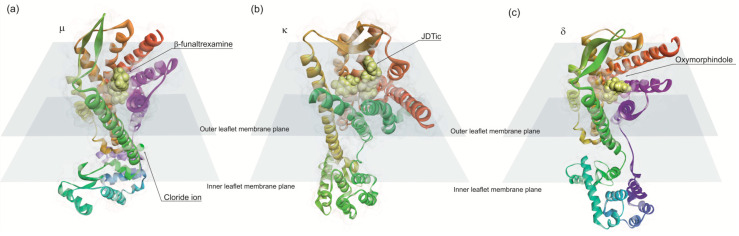
Opioid Receptors (**a**) µ (PDB: 4dkl), (**b**) κ (PDB: 4djh) and (**c**) δ (PDB: 4ej4). Theses receptors exhibit seven transmembrane helices, typical of G-protein-coupled receptor (GPCR) structures. The binding hydrophobic pocket accommodates the aliphatic substituents on the amino group and the phenolic group of morphine engages an extended hydrogen-bonding network between two water molecules and a conserved histidine residue in transmembrane helix 6 (TM6).

Opioid binds to G protein-coupled (Gi and/or Go) receptors [234,235]. The opioid receptors are present in CNS and are classified into four types: µ, κ, δ and nociceptin [236]. µ-receptors mediate natural rewards initiating addictive behaviors [237], whereas δ and κ-receptor activity appears to play a role in improving mood states [238,239]. These receptors bind to endogenous and exogenous opioids structurally related to the natural plant alkaloids found in opium, but also to small opioid peptides. Nociceptin receptor binds to medium size endogenous opioid peptides such as nociception and orphanin. Theses receptors exhibit seven transmembrane helices, typical of GPCR structures. The atomic structures of opioid receptors revealed common features for opioid recognition as predicted previously [240,241,242]. Opioid receptors binding sites contain an anionic aspartic acid residue that forms an ionic bond with the amino group of opioid ligands (Figure 5). The binding hydrophobic pocket accommodates the aliphatic substituents on the amino group and the phenolic group of morphine engages an extended hydrogen-bonding network between two water molecules and a conserved histidine residue in transmembrane helix 6 (TM6) [243]. The activation of the receptor is due to a conformational change displacing TM6 10 A and, to a lesser extent, TM5 and TM7. These movements open a large pocket in the intracellular side of the receptor allowing to couple the heterotrimeric G proteins [243].

After binding to these receptors, opioids inhibit voltage-dependent Ca^2+^ channels or activate inwardly rectifying potassium channels, thereby diminishing neuronal excitability [234]. Opioids also inhibit the cyclic adenosine monophosphate pathway and activate mitogen-activated protein kinase cascades, both of which affect cytoplasmic events and transcriptional activity of the cell [234]. Overall, opioids inhibit neurons by decreasing either neuronal firing on the postsynaptic localization or neurotransmitter release on presynaptic localization of the receptors. Finally, since opioid receptors are expressed on both excitatory and inhibitory neurons, they can exert activation or inhibition of the neural circuits [234]. In addition to opioids, opioid peptides are sharing a common N-terminal Tyr-Gly-Gly-Phe signature sequence that also interact with opioid receptors, namely β-endorphin, enkephalins and dynorphins which bind to µ, δ and κ, respectively [234].

In the rat model of PD, studies suggest the involvement of opioid pathways in the mechanisms modulating nociceptive thresholds [244]. Another study observed an increase in the survival rate of dopaminergic neurons treated with δ opioid peptide, when exposed to the neurotoxin 6-OHDA, both in vitro and in vivo [245]. These results suggest that δ-opioid receptors may be protective in PD [245]. Moreover, more studies performed in rat and primate models of PD indicates that δ-opioid receptors reduce dyskinesia induced by levodopa [246,247].

Regarding endogenous opioids, it is now accepted that endogenous morphine, structurally similar to vegetal morphine-alkaloid, is synthesized by mammalian cells from dopamine [248]. It binds to µ opioid receptor and induces antinociceptive effects. In PD patients the levels of endogenous morphine and its metabolites were increased [249]. This increment may be associated with fatigue, depression and pain symptoms experienced by PD patients [249]. Opioids affect locomotion and reward behavior mediated by the basal ganglia [250,251]. Since the striatum is rich in both μ-and δ-opioid receptors, these substances can act as modulators of dopamine, gamma-aminobutyric acid (GABA) and glutamate neurotransmission [250,252].

An increase in opioid transmission in the two main striatal outputs has been observed in monkeys or humans with dyskinesis induced by levodopa, which may indicate that the endogenous opioid system must be involved in mitigating the effect of abnormal dopaminergic stimuli. This knowledge can help to find therapeutic strategies for the treatment and prevention of motor complications in PD [253]. On the other hand, prolonged treatment with oxycodone-naloxone seems to affect only specific subgroups of PD patients with pain, which suggests that successful clinical improvements require a careful identification and characterization of PD patients [254].

Cellular studies showed that δ-receptor activation attenuates α-synuclein expression and aggregation reducing cytotoxicity in vitro PD model exposed to MPP(+) stress [255]. δ-receptor activation can largely attenuate α-synuclein expression via DJ-1 upregulation in both genetic (α-syn wild-type or A53T-mutant α-syn) and environmental (hypoxic) conditions. Moreover, the δ-receptor action involves transducer of regulated CREB1 (TORC1) / salt-inducible kinase 1 (SIK1) downregulation in the former condition and cAMP response element-binding protein (CREB) phosphorylation in the latter condition [256]. The activation of δ-receptor seems to be cytoprotective against both hypoxia and MPP+ through the regulation of PTEN-induced kinase 1 (PINK1) and caspase 3 pathways [257] Although, activation of δ-receptors has anti-parkinsonian effect, adverse effects of opioids were also observed. A long-term exposure to tramadol is known to induce tremor, muscular rigidity and tardive dyskinesia [258]. These symptoms are possibly related to: (i) serotonin’s inhibitory effect on dopamine neurotransmission within the basal ganglion system, which may result in the altered function in the striatum [259]; and (ii) the inhibition of serotonin reuptake inhibitors [260].

### 6.1. Morphine and Parkinson’s Disease

Morphine is a partial agonist for μ-opioid receptors and acts as a weak agonist for δ-opioid receptors. However, morphine does not seem to act through κ-opioid receptors [236]. It was suggested that morphine raises dopamine levels in the brain by stimulating µ opioid receptors, which inhibit GABA release and consequently enhances dopamine release [261,262]. Therefore, µ opioid receptors are a potential therapeutic target in PD symptom relief. This is reinforced by clinical observations showing that morphine alleviates tremor significantly [263]. However, the levels of α-syn protein in mice withdrawn from morphine for 48 h were significantly increased in the ventral striatum, namely nucleus accumbens and two weeks after treatment cessation the protein levels were still high [264].

According to Fan et al. (2019), morphine increases the cell viability in PC12 cells after MPP^+^ exposition. MPP^+^ reduces cell viability and tyrosine hydroxylase expression, but this effect was reversed in the presence of morphine which acts on the P13K/Akt pathway [265]. Moreover, it was shown that morphine have neuroprotective effects against 6-OHDA-induced SH-SY5Y dopaminergic cell damage and neurodegeneration [266,267]. Moreover, it was shown that morphine contributes for Ca^2+^ homeostasis and ROS production decreasing in 6-OHDA-treated SH-SY5Y cells [266].

Morphine potentially changes the expression of PD-associated genes. Mantione (2014) reported that PARK2 was up-regulated and PINK1 was down-regulated [268]. These two genes are associated with juvenile PD. Mutations in PARK2 are the cause of near 50% of autosomal recessive juvenile Parkinsonism [269]. PINK 1 overexpression activates Parkin’s E3 ubiquitin ligase and recruits Parkin triggering selective autophagy [270,271].

### 6.2. Heroin

Heroin is a semisynthetic product obtained by acetylation of morphine, which occurs as a natural product in opium, the dried latex of certain poppy species (e.g., *Papaver somniferum* L.). Heroin is a narcotic analgesic used in the treatment of severe pain. This substance crosses the blood-brain barrier within twenty seconds and almost 70% of the dose reaches the brain after injection. Heroin is 2–3 fold more potent than morphine. It is difficult to detect in the blood since it is rapidly hydrolyzed to 6-monoacetylmorphine and slowly converted to morphine, the main active metabolite.

Heroin is the most common opioid on the European Union drug market and, in 2017, it was second in the rank of drugs responsible for emergency attendance in hospitals [170]. Like other opioids, this drug of abuse interacts with opioid receptors localized in the peripheral and central nervous system. Heroin is usually injected or smoked. The inhalation of the vapor, resulting from heroin heated on aluminum foil (*chasing the dragon*) has been associated with spongiform encephalopathy [272,273,274,275,276,277,278,279,280] and Parkinsonism [47,48]. However, despite of a case report describing temporary Parkinsonism in a patient who inhaled heroin vapor, it was found a reversible deficiency of tetrahydrobiopterin underlying the altered dopamine metabolism [48]. Another case report refers that, twenty four hours after snorting heroin, a patient exhibited a generalized dyskinetic syndrome and impaired vision, and severe parkinsonian symptoms which have worsened in the following two weeks [281]. More cases were reported, however, in all these cases the heroin was analyzed and contaminants were found, namely MPTP, 1-methyl-4-phenyl-4-propionoxypiperidine (MPPP) (1-methyl-4-phenyl-4-propionoxypiperidine) and 1-methyl-4-phenylpyridinium ion (MPP+) [14,282,283]. The relationship between these contaminants and PD has already been described. MPPP, a heroin analogue, is quickly converted in its metabolite MPP^+^, which promotes a syndrome indistinguishable from Parkinsonism, after cell uptake by the dopamine transporter of dopaminergic neurons inhibiting the activity of the mitochondrial nicotinamide adenine dinucleotide hydride (NADH)-Q dehydrogenase complex (EC 1.6.5.3) [284,285,286,287]. Moreover, MPTP also destroys dopamine-making cells in *substantia nigra* [283]. In addition to the contaminants present in heroin preparation, this drug is normally combined with other substances of abuse, namely mephedrone [282]. In this context, there are no molecular evidence that heroin is associated with PD onset and the case reports are unclear, as heroin is mostly consumed with contaminants.

## 7. Future Issues: Novel Psychoactive Substances-Protective or Neurotoxic?

The emergence of NPS over the last decade has been challenging drug policies [288]. An NPS is defined as “a new narcotic or psychotropic drug, in pure form or in preparation, that is not controlled by the United Nations drug conventions, but which may pose a public health threat comparable to the substances of abuse listed in these conventions” [289]. NPS have been emerging in the street market and on the Internet on a regular basis. Their properties change regularly, due to structural modification to circumvent legislation. This practice makes it almost impossible to characterize its toxicological profiles on an acceptable time scale, mostly due to the time-consuming experiments that must be held in animal models or human cells by standard methods [290]. NPS are associated with deaths and acute intoxications in Europe, as well as changes to current drug policy models [170]. NPS are classified in several groups, of which the most consumed are synthetic cannabinoids and synthetic cathinones [170].

### 7.1. Synthetic Cannabinoids

Cannabinoid receptor agonists have been developed with therapeutic purposes. However, most of these substances were not approved by medicine regulatory agencies. These substances were hijacked and misused for recreational purposes [291]. Synthetic cannabinoids are a group of NPS with similar properties to ∆^9^-THC that appeared in the drug market in 2004 as a herbal blend [288]. Since then, the composition of these cannabinoid containing herbal products has substantially changed to include more potent new psychoactive compounds and circumvent the law. Synthetic cannabinoids are divided into several classes which JWH series are among the oldest. These synthetic cannabinoids are representative of scientific research which the illegal market turned them into potent, dangerous recreational drugs that strongly activate the brain reward pathway and expose the user to a higher risk of developing addiction and other severe illnesses, including psychosis [292].

In vivo studies showed that synthetic cannabinoids 2-(2-methoxyphenyl)-1-(1-pentyl-1H-indol-3-yl)ethenone (JWH-250) and 1-butyl-3-(1-naphthoyl)indole (JWH-073), major agonists of CB1, induced several effects when administered separately, such as impaired sensorimotor responses (visual, acoustic and tactile), caused seizures, myoclonia, hyperreflexia and promote aggressiveness in mice [293]. However, a co-administration of ineffective doses of JWH-250 and JWH-073 impaired visual stimulated mesolimbic dopamine transmission in mice. Such experiments have shown the potential synergistic action of synthetic cannabinoids suggesting that co-administration of these NPS may potentiate the harmful effects of individual compounds increasing their dangerousness [293].

Recently, the impact of 1-pentyl-3-(1-naphthoyl)indole (JWH-018), a more potent agonist of CB1 than ∆^9^-THC, was described at the molecular and cellular level, using yeast as a eukaryotic model [294]. In the presence of JWH-018, the cells’ growth rate is higher due to an enhanced glycolytic flux at expenses of a decrease in pentose phosphate pathway (PPP) [294]. PPP generates NADPH, which is critically important since its production provides reducing power to deal with the oxidative stress [295]. In the brain, reactive oxygen species come mainly from dopamine metabolism, mitochondrial dysfunction and neuroinflammation, and its oxidative stress contributes to PD [296]. Consequently, the reduction of NADPH production during a long period might allow the proliferation of reactive oxygen species, thus increasing the probability of developing PD in JWH-018 consumers. However, further studies must be performed to understand such implications in PD models.

Despite the possible neurotoxic effects of synthetic cannabinoids, (6aR,10aR)-6,6,9-trimethyl-3-(2-methylpentan-2-yl)-6a,7,10,10a-tetrahydrobenzo[c]chromene (JWH-133), a potent agonist of CB2, showed to be neuroprotective in a MPTP model of PD. This response was associated with the suppression of blood-brain barrier damage, astroglial myeloperoxidase expression, infiltration of peripheral immune cells and production of inducible nitric oxide synthase, proinflammatory cytokines and chemokines by activated microglia [127].

In synthetic cannabinoids, the affinity to CB1 or CB2 seems to be essential in understanding if a compound might be neurotoxic or protective to PD, respectively.

### 7.2. Synthetic Cathinones

Synthetic cathinones appeared in drug markets in the mid-2000s [288]. They are derived from cathinone, which is the principal active ingredient in the leaves of the khat plant (*Catha edulis*). Since cathinones are structurally similar to amphetamine, it has been hypothesized that these substances also share the same mechanisms of action. Synthetic cathinones are divides into two major groups according to its effects. On group includes cathinones with amphetamine-related pharmacological activities, lacking the methylenedioxy ring, and another group comprises those with effects similar to “ecstasy”, bearing the methylenedioxy ring [297]. Mephedrone is a synthetic cathinone causing Parkinson type symptomatology (in the form of spasms and ‘wobbling’) [282]. This powerful stimulant seems to work as a monoamine reuptake inhibitor, increasing serotonin, norepinephrine and dopamine levels at neuronal synapses, which leads to dangerous neurological complications, such as reversible encephalopathy [298,299,300]. Mephedrone is normally combined with other drugs of abuse, and the literature indicates that it significantly enhances the neurotoxicity to dopamine nerve endings of the striatum caused by methamphetamine, amphetamine and 3,4-methylenedioxymethamphetamine (MDMA), showing that the consumption of these mixtures may still represent a greater risk [301]. Moreover, a recent study conducted in human differentiated neuronal cells revealed that amphetamine does not generate significant amounts of reactive oxygen species compared to the negative control. However, the cells showed significant levels of reactive oxygen species in the presence of 3,4-dimethylmethcathinone (3,4-DMMC), methcathinone and pentedrone [302], meaning that these new compounds could be more dangerous than the natural cathinone. In addition, the reactive oxygen species production by cells in the presence of these compounds might be a risk factor for developing PD. Furthermore, neurodegenerative effects have also been observed. A recent study with methylenedioxypyrovaleron (MDPV) proved that long-term use increases the risk of impaired cognitive function and neurodegeneration in the prefrontal cortex or hippocampus [303]. In sum, synthetic cathinones appear to induce neurocognitive dysfunction and cytotoxicity, which are dependent on drug type, dose, frequency and time following exposure [304].

## 8. Synthesis of the Available Data on Illicit Drugs and Parkinson’s Disease

The study of illicit substances’ effects on PD combines data from basic, clinic and epidemiological research. Table 1 abridge all the scientific data previously exposed, aiming to understand whether drugs of abuse are neuroprotective or neurotoxic regarding PD.

Phytocannabinoids are used legally or illegally by PD patients worldwide despite their use have not been approved by the EMA and FDA for PD. In general, the effects of phytocannabinoids on PD appear to be protective either by binding to the CB1 receptor or by CB2. As an example, the bind of ∆^9^-THC to CB1 restores membrane potential; decrease ROS; increase CB1 protein level which will increase the amount of synaptic vesicles inhibition. Moreover, after a decrease of the mitochondrial potential membrane by MPP+ exposition, the activation of CB2 might be neuroprotective by inhibiting the apoptosis by Trka/NGF.

The effects of amphetamine-type stimulants consumption in PD is fairly scientifically documented. The effects of amphetamines seem to be neurotoxic and the several molecular and cellular interaction of amphetamines-type substances seems to result in the aggregation or in the increment of ROS by dysregulated cellular Ca^2+^ which activate nitric oxide synthetase or dopamine oxidation. Cocaine is also a stimulant, but studies do not seem to point a protective or neurotoxic effect of cocaine. However, there are few studies suggesting that cocaine might be neurotoxic promoting PD by increasing α-syn levels and consequently its aggregation.

Morphine is vastly used in a medical context, mostly as an analgesic. It is suggested that morphine is neuroprotective in PD by increasing the brain dopamine levels, stabilizing Ca^2+^ homeostasis, decreasing ROS production and altering the expression of PD-associates genes, which counteract the neurodegeneration triggers.

## 9. Conclusions

If drugs of abuse are neuroprotective or neurotoxic regarding PD onset is highly dependents on the type of substance and is still a matter of debate. However, the evidence obtained by different research groups have been pointing to similar effects for the same class of illicit substances. Concerning cannabinoids, evidence from basic and clinical research along with epidemiological studies suggest that phytocannabinoids have the potential to prevent and alleviate PD. Notwithstanding, some results are inconsistent. Discrepancies are partially explained by differences in research methodologies and by the translation of data gathered in animal models to humans. Actually, animal models do not recapitulate the timeline of PD pathology in humans. The effects of amphetamine-type stimulants consumption in PD seem to be better scientifically documented from basic to clinical research and epidemiological evidence. In general, these studies associate amphetamine stimulants consumption to PD onset. Regarding opioids, the basic, clinical and epidemiological studies suggest that they are neuroprotective in PD. Opioids regulate levels of dopamine, calcium and ROS, which counteract the neurodegeneration triggers. Although heroin was associated with PD clinical observations, the scientific studies do not support this association, and the evidence point out heroin cutting agents as the cause of PD. The impact of NPS consumption in PD is yet to be revealed since it is a recent trend. However, the few scientific results in the literature point to neurotoxicity and the hypothesis of these substances being implicated in PD onset cannot be discarded.

To sum up, phytocannabinoids and morphine seem to be neuroprotective while amphetamine-type stimulants seem to be neurotoxic. In addition, there is not enough data to support the involvement of cocaine and heroin in PD. Overall, this review gathers current knowledge on the relationship between illicit drugs and PD, which is utterly important for contemporary society, as illicit drug legalization is under discussion in many countries worldwide, and the health consequences must be balanced.

## Figures and Tables

**Table 1:** The effect and mechanism of action of illicit drugs of abuse on PD as described in the literature.

Class of Substance	Substance	Mechanism of Action Related to PD	Neurotoxic or Neuroprotector	Ref
**Phytocannabinoids**	Δ^9^-THC	upregulates the expression of gene encoding CB1	Neuroprotector	[55]
		induce the transcription of proteins involved in oxidative stress defense and mitochondrial biogenesis, promoting mitochondrial normal function		[151]
		expresses mitochondria transcription factors (TFAM) and restore mitochondrial DNA levels leading to increased cytochrome c oxidase subunit 4 (COX4)		[151]
		effective against glutamate-induced neurotoxicity restoring mitochondrial membrane potential which produces an anti-apoptotic effect.		[154]
	cannabidiol	effective against MPP+ neurotoxin by the activation of NGF/TRKA receptors and the increment in expression of axonal and synaptogenic proteins	Neuroprotector	[155]
	β-caryophyllene	decreases oxidative/nitrosative stress, decrease pro-inflammatory cytokines release and to an inhibition of gliosis	Neuroprotector	[156,157]
	Δ^9^-THCV	acute administration changes glutamatergic transmission, and the chronic administration was shown to reduce the loss of tyrosine hydroxylase–positive neurons caused by 6-hydroxydopamine in the *substantia nigra*	Neuroprotector	[158]
**Stimulants**	Amphetamine and methamphetamine	bind tightly to N-terminus of intrinsically unstructured α-syn adopting a folded conformation, increasing the likelihood of misfolding	Neurotoxic	[189,190]
	Amphetamine and methamphetamine	involvement of tyrosine hydroxylase, dopamine transporter and vesicular monoamine transporter 2 in the decrease of dopamine levels	Neurotoxic	[203]
	methamphetamine	increments α-syn levels induced by excessive heat	Neurotoxic	[191]
		causes post-translational modification of α-syn by nitration increase expression of nT39 α-syn.		[192]
		decreases cytosine methylation in SNCA promoter region, and consequently upregulates α-syn in the in *substantia nigra*		[199]
		activates nicotinic alpha-7 receptors, which increase intra-synaptosomal calcium, nitric oxide synthase and protein kinase C, leading to the production of unjustified nitric oxide and dopamine oxidation		[200]
		induces higher levels of oxidative stress as a consequence of dopamine autoxidation and increasing excitotoxicity as a result of perturbations in energy metabolism.		[205]
		low doses induce the expression of a different set of genes in lesioned denervated striatum, completely lacking dopamine (i) decreases basal ERK 1/2 and kinase b levels, involved in multiple cellular processes such as apoptosis; (ii) reduces the activity of protein phosphatase 2, a protein phosphatase implicated in ERK1/2 dephosphorylation, inhibiting it; and (iii) upregulates the pro-survival protein BCL-2, which plays an anti-apoptotic role	Neuroprotector	[196]
	Cocaine	binds tightly to N-terminus of intrinsically unstructured α-syn adopting a folded conformation, increasing the likelihood of misfolding	Neurotoxic	[189]
		increments α-syn levels		[225,226,227]
**Opioids**	Morphine	elevates brain dopamine levels by stimulating µ opioid receptor, which inhibits GABA release and consequently enhances dopamine release	Neuroprotector	[261,262]
		reverses MPP+ toxicity through activating P13K/Akt pathway		[265]
		stabilizes Ca^2+^ homeostasis and decreases ROS production and cytochrome c in 6-OHDA-treated cells.		[266,267]
		alters PD-associated genes expression, whereas PARK2 is up-regulated and PINK1 is down-regulated.		[268]
**Synthetic cannabinoid**	JWH-018	enhances glycolytic flux at expenses of a decrease in pentose phosphate pathway	Neurotoxic	[294]
	JWH-133	suppresses blood–brain barrier damage, astroglial myeloperoxidase expression, infiltration of peripheral immune cells and production of inducible nitric oxide synthase, proinflammatory cytokines and chemokines by activated microglia	Neuroprotector	[127]
**Synthetic cathinone**	mephedrone	monoamine reuptake inhibitor, increasing serotonin, norepinephrine and dopamine levels at neuronal synapses	Neurotoxic	[298,299,300]
	3,4-DMMC, methcathinone and pentedrone	Increases the levels of reactive oxygen species	Neurotoxic	[302]
